# Genome-Wide Integrated Analysis Revealed Functions of lncRNA–miRNA–mRNA Interaction in Growth of Intermuscular Bones in *Megalobrama amblycephala*

**DOI:** 10.3389/fcell.2020.603815

**Published:** 2021-02-04

**Authors:** Yulong Chen, Shiming Wan, Qing Li, Xiaoru Dong, Jinghan Diao, Qing Liao, Gui-Ying Wang, Ze-Xia Gao

**Affiliations:** ^1^Key Lab of Agricultural Animal Genetics, Breeding and Reproduction of Ministry of Education, Key Lab of Freshwater Animal Breeding, Ministry of Agriculture, College of Fisheries, Huazhong Agricultural University, Wuhan, China; ^2^Engineering Research Center of Green Development for Conventional Aquatic Biological Industry in the Yangtze River Economic Belt, Ministry of Education, Wuhan, China; ^3^Fisheries Research Institute, Wuhan Academy of Agricultural Sciences, Wuhan Xianfeng Aquaculture Technology Co. Ltd, Wuhan, China; ^4^Engineering Technology Research Center for Fish Breeding and Culture in Hubei Province, Wuhan, China

**Keywords:** teleost, intermuscular bones, growth, transcriptome profiling, RNA interaction

## Abstract

Intermuscular bone (IB) occurs in the myosepta of teleosts. Its existence has an adverse influence on the edible and economic value of fish, especially for aquaculture species belonging to Cypriniformes. The growth mechanism of IBs is quite lacking. In this study, we firstly used single molecular real-time sequencing (SMRT) technology to improve the draft genome annotation and full characterization of the transcriptome for one typical aquaculture species, blunt snout bream (*Megalobrama amblycephala*). The long non-coding RNA (lncRNA), microRNA (miRNA), and messenger RNA (mRNA) expression profiles in two IB growth stages (1 and 3 years old) were compared through transcriptome and degradome analyses. A total of 126 miRNAs, 403 mRNAs, and 353 lncRNAs were found to be differentially expressed between the two stages. Kyoto Encyclopedia of Genes and Genomes (KEGG) analysis revealed that the significantly upregulated *map2k6* and *cytc* in the MAPK/p53 signaling pathway and the significantly downregulated *lama3* and *thbs4b* in the extracellular matrix (ECM)–receptor pathway may play a key regulatory role in IB growth. Bioinformatics analysis subsequently revealed 14 competing endogenous RNA (ceRNA) pairs related to the growth of IBs, consisting of 10 lncRNAs, 7 miRNAs, and 10 mRNAs. Of these, dre-miR-24b-3p and dre-miR-193b-3p are core regulatory factors interacting with four lncRNAs and three mRNAs, the interaction mechanism of which was also revealed by subsequent experiments at the cellular level. In conclusion, our data showed that IBs had higher activity of cell apoptosis and lower mineralization activity in IB_III compared to IB_I via interaction of MAPK/p53 and ECM–receptor signaling pathways. The downregulated *zip1* interacted with miR-24a-3p and lnc017705, decreased osteoblast differentiation and Ca^2+^ deposition in the IB_III stage. Our identified functional mRNAs, lncRNAs, and miRNAs provide a data basis for in-depth elucidation of the growth mechanism of teleost IB.

## Introduction

Intermuscular bone (IB), which only occurs in the myosepta of lower teleosts, is a small spicule-like bone derived from tendons (Danos and Ward, 2012). Most freshwater-farmed fishes have a substantial amount of IB, which has an extremely negative impact on their edible and economic value. Earlier studies have revealed the number and complex morphological characteristics of IB in different species (Dong et al., 2006; Wan et al., 2014). Subsequent research also revealed the possibility of reducing IB numbers based on ploidy change (Li et al., 2013), distant hybridization (Jiang et al., 2016), and genetic breeding (Cao et al., 2015; Xiong et al., 2018). It is worth noting that an IB-deficient grass carp (*Ctenopharyngodon idellus*) mutant and some specimens of tambaqui (*Colossoma macropomum*) without IB (normal individuals possessing significant numbers of IB) were found in an artificial gynogenetic group and a culture population (Xu et al., 2015; Perazza et al., 2016), respectively, which strongly indicated the feasibility of genetic improvement of IB numbers. Therefore, clarification of the regulatory mechanism of IB growth will contribute to trait improvement in aquaculture.

The biological phenotypic diversity was dominated by complex molecular mechanisms involving DNA modification and the regulation of transcription and translation, in which long non-coding RNA (lncRNA) and microRNA (miRNA) also play important roles through regulating gene expression. Numerous studies have paid attention to the biological function of lncRNA and revealed its important roles in cell differentiation, transcription regulation, and development (Yang et al., 2014; Larsson et al., 2015; Liu et al., 2015; Ali and Soudeh, 2017). In fish, lncRNA has also been reported to participate in many biological regulatory processes, including early sex differentiation (Cai et al., 2019; Yuan et al., 2019), skin color regulation (Luo et al., 2019), and immune regulation (Wang M. et al., 2018). miRNA can regulate transcript levels by binding the 3′ untranslated region (UTR) of the target mRNA to participate in various biological processes, such as osteoblast differentiation, development, disease, gene transcription, and translation (Hobert, 2008). So far, the miRNA-related studies had been reported in several fishes including zebrafish (*Danio rerio*), blunt snout bream (*Megalobrama amblycephala*), *Botia superciliaris*, and *Pelteobagrus vachelli*. These studies mainly pay attention to the regulatory role of miRNA in the growth (Zhao et al., 2017) and development of organs including the skeleton, gonad, skin, and liver (Gan et al., 2016; Zhang et al., 2016; Lan et al., 2019; Zhou et al., 2019). Nonetheless, there are few miRNA studies on fish IB development. In our previous studies, the dynamic expression patterns and potential function of mRNA and miRNA within IB development were revealed in the typical aquaculture species blunt snout bream (Wan et al., 2016). Meanwhile, miRNA transcriptome features of IB and surrounding connective tissues had also been investigated in *M. amblycephala* (Wan et al., 2015), which provided theoretical support for revealing the potential molecular regulatory mechanisms of IB development. Massive evidences had shown that lncRNA and miRNA play an important regulatory role in animal bone development, but the specific regulatory function of lncRNA and miRNA in fish IB development is still unclear.

The single molecular real-time sequencing (SMRT) technology can provide important information for improving draft genome annotation and the full characterization of the transcriptome for corresponding species (Sharon et al., 2013; Gao et al., 2016). This technology has also been widely used in fishes, by which Gong et al. (2018) constructed the first high-quality chromosome-level genome assembly in yellow catfish (*Pelteobagrus fulvidraco*), which offered a valuable reference for functional genomics studies of yellow catfish to decipher its economic traits and sex determination. The genome assembly and gene evolution analysis of goldfish (*Carassius auratus*) also provide an important resource for understanding the causes of goldfish variants (Chen et al., 2017). With the development of high-throughput sequencing technology, transcriptome sequencing has been utilized in various fields and species. However, the regulatory networks that determine specific traits are often extremely complex; it is difficult to accurately discover key regulatory factors from a single omics data. Fortunately, the development of multi-omics research has overcome this shortcoming, and the competing endogenous RNA (ceRNA) research has also become a new hot spot in revealing the mechanism of RNA interaction.

**Figure 1:**
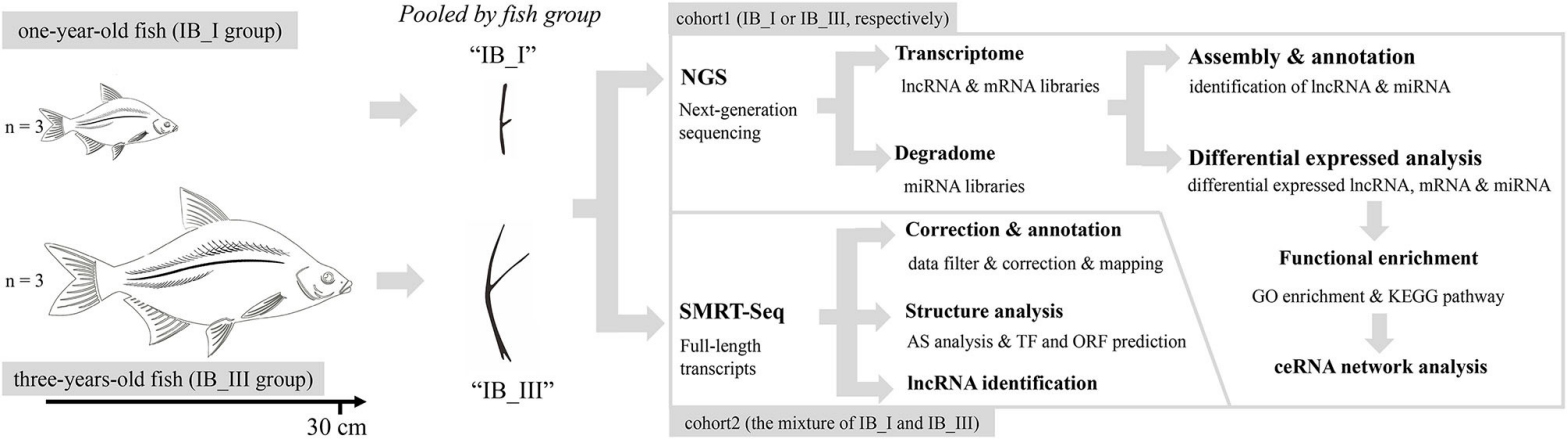
Phenotypic characteristics of IB and research scheme diagram.

In this study, we firstly used SMRT technology to improve the draft genome annotation and full characterization of the transcriptome for *M. amblycephala*. Then, the lncRNA, mRNA, and miRNA expression profiles of IB samples in 1- and 3-year old specimens were revealed, respectively. Based on conjoint analysis of multi-omics at different periods, the lncRNA–miRNA–mRNA ceRNA regulatory network related to IB growth was detected and verified. This research aims to reveal the key lncRNA, miRNA, and target mRNA involved in teleost IB growth and provides an important reference for further revealing the molecular regulatory mechanism of IB growth (Figure 1).

## Materials and Methods

### Experiment Materials and Sample Preparation

Before sampling, experimental *M. amblycephala* were anesthetized in well-aerated water containing the 100 mg/L concentration of tricaine methanesulfonate. IB samples of three 1-year-old (IB_I, IBs growing rapidly at this stage) and three 3-year-old (IB_III, IBs growing quite slowly at this stage) fishes were collected and pooled, respectively, with three biological replicates. Total RNA was isolated using RNAiso Plus Reagent (Takara, Japan) following the manufacturer's protocol. RNA degradation and contamination were monitored on 1% agarose gels. RNA concentration was measured using the NanoDrop 2000 spectrophotometer. Qualified RNA samples are stored at −80°C and then used for RNA sequencing. All animals and experiments were conducted in accordance with the “Guidelines for Experimental Animals” of the Ministry of Science and Technology (Beijing, China). The study was approved by the Institutional Animal Care and Use Ethics Committee of Huazhong Agricultural University. All efforts were made to minimize animal suffering.

### Library Construction and Sequencing

An equal mixture of RNA from all samples of two periods was used for the Pacific Bioscience (PacBio) library construction. The mRNA containing polyA was enriched with Oligo(dT), which was then reversely transcribed into cDNA using the Clontech SMARTer PCR cDNA synthesis kit. After PCR amplification, DNA damage repair, end repair, SMRT adapter ligation, size fractionation, and selection (<4 and >4 kb), two SMRT bell libraries were constructed, and the combined SMRT bell library was then sequenced on the PacBio Sequel System.

lncRNA and small RNA sequencing libraries were generated using NEBNext® Ultra™ Directional RNA Library Prep Kit (NEB, USA) and NEBNext® Multiplex Small RNA Library Prep Set (NEB, USA), respectively, following the manufacturer's recommendations. The PCR products were purified (AMPure XP system), and library quality was assessed on the Agilent Bioanalyzer 2100 system. After cluster generation of the index-coded samples using TruSeq PE Cluster Kit v3-cBot-HS (Illumina), the libraries were sequenced on an Illumina HiSeq 2500 platform. Then 125 bp paired-end reads and 50 bp single-end reads were generated in lncRNA and small RNA libraries, respectively.

### Correction, Annotation, and Structural Analysis of Full-Length Transcripts

The adaptor and raw reads with lengths <50 bp were removed to generate subreads, which subsequently were processed using the SMRT Link software to generate a circular consensus sequence (CCS). Subsequently, the full-length non-chimeric (FLNC) reads with complete 5′ primer, 3′ primer, and poly(A) tails of the same transcript were clustered using the iterative clustering for error correction (ICE) algorithm to remove redundancy and obtain consensus reads, and the non-full-length (NFL) sequences were further used to correct consensus reads using the arrow algorithm to obtain polished consensus reads, which were further corrected and improved via the Illumina RNA-Seq data (NCBI BioProject: PRJNA329421) generated by our lab and LoRDEC software (Salmela and Rivals, 2014). Later, corrected polished consensus sequences were aligned to the reference genome (Liu et al., 2017) using the genome mapping and alignment program (GMAP) (Wu and Watanabe, 2005). According to the genome mapping results, the polished consensus sequence was further corrected and clustered to remove redundancy and obtain the final high-quality isoforms using the TAPIS pipeline (Abdel-Ghany et al., 2016).

SUPPA (Alamancos et al., 2015) was used to identify seven types of alternative splicing (AS) events: skipped exon (SE), mutually exclusive exon (MX), alternative 5′ splice site (A5), alternative 3′ splice site (A3), retained intron (RI); alternative first exon (AF); and alternative last exon (AL). In order to identify poly(A) sequence signals and transcription factors (TFs) in our PacBio Iso-Seq data, MEME analysis (Zhang et al., 2015) and Animal Transcription Factor Database (AnimalTFDB) analysis were performed, respectively. In order to predict open reading frames (ORFs), the ANGEL pipeline (Kana et al., 2006) was used to find potential coding sequences for corrected isoforms. Meanwhile, PLEK (Lei et al., 2007), CNCI (Li et al., 2014), CPC (Sun et al., 2013) software, and Pfam (Finn et al., 2016) databases were used to predict the coding potential of PacBio sequencing data to identify lncRNA. At last, unmapped transcripts and novel gene transcript functions were annotated based on the following databases: NCBI non-redundant protein sequences (NR); NCBI non-redundant nucleotide sequences (NT); Pfam (protein family), KOG/COG (Clusters of Orthologous Groups of proteins (KOG/COG); Swiss-Prot (a manually annotated and reviewed protein sequence database); Kyoto Encyclopedia of Genes and Genomes (KEGG); and Gene Ontology (GO).

### Assembly and Annotation of lncRNA and miRNA Transcriptomes

After quality control, paired-end clean reads were aligned to the reference genome using HISAT2 (Langmead and Salzberg, 2012). The mapped reads of each sample were assembled by StringTie (Mihaela et al., 2016). After transcripts that overlapped with known mRNAs or shorter than 200 bp were discarded, the coding potential analysis was performed on the remaining transcripts to define candidate lncRNA by CPC2 (Kang et al., 2017), CNCI (Li et al., 2014), and Pfam (Finn et al., 2016). Target gene prediction of lncRNA was then performed, and the expression level of both lncRNAs and coding transcripts were quantified.

The small RNA tags were mapped to the reference sequence by Bowtie (Langmead et al., 2009) without mismatch to analyze their expression and distribution on the reference. Mapped small RNA tags were used to look for known miRNAs based on the miRBase 20.0 database and miRDeep2 (Friedländer et al., 2012) and to predict novel miRNAs by miREvo (Wen et al., 2012) and miRDeep2. The target gene prediction of miRNA was then performed by miRanda (Enright et al., 2003), and miRNA expression levels were estimated.

### Differential Expression and Functional Enrichment Analyses

A comparison of expression levels of mRNA, lncRNA, and miRNA between IB_I and IB_III was performed using DESeq or edgeR (Robinson et al., 2010). Differentially expressed mRNAs/lncRNAs/miRNAs were screened with a threshold *q* < 0.05. The lncRNA target genes were predicted by co-location analysis within 100 kb upstream and downstream of the lncRNA. And co-expression analysis was also used for lncRNA target prediction based on the Pearson correlation coefficient method with a threshold *P* < 0.05 and a correlation absolute value (*R*^2^) >0.95. Besides, GO and KEGG pathway enrichment analyses of differentially expressed genes or lncRNA target genes were implemented by the GO-seq R package (Young et al., 2010) and KOBAS software (Mao et al., 2005), respectively.

### ceRNA Analysis

Pearson correlation coefficients of lncRNA and mRNA were calculated using the corresponding matrix data, and then a correlation test was performed. According to the ceRNA mechanism, significant positively correlated co-expression of lncRNA–mRNA was screened under conditions of correlation coefficient *r* > 0.95 and *P* < 0.001. The lncRNA–miRNA and mRNA–miRNA targeted pairs were constructed based on the targeting relationship between differential expressions of mRNA, lncRNA, and miRNA. The ceRNA regulatory network (lncRNA–miRNA–mRNA) was constructed based on the co-targeted lncRNA–miRNA–mRNA pairs and co-expressed lncRNA–mRNA pairs, followed by visualization of the ceRNA regulatory network using the Cytoscape software.

### Validation of Transcriptome Sequencing

For validation of AS isoforms, five isoform genes were randomly selected, and gene-specific primers were designed using NCBI Primer-BLAST, and the expression profiles of nine lncRNAs and nine miRNAs were verified using qPCR technology. The PCR products of AS isoforms mixed with 10 × loading buffer and GelRed fluorescent nucleic acid dyes were electrophoresed in 1.4% agarose gel for 1 h. Real-time qPCR was performed to validate gene expression using TB Green® Fast qPCR Mix on the QuantStudio™ 6 Flex qRT-PCR system (ABI, Germany). The specific primer pairs of the validated genes were shown in Supplementary Table 1. The relative expression of the genes was calculated based on the comparative CT (2^−ΔΔCT^) method. Statistical analysis was performed using SPSS software. Data were presented as mean ± standard deviation (SD), with a statistical significance of *P* < 0.05.

### Vector Construction

The whole lengths of lnc017705 and the dre-miR-24b-3p gene were cloned into the overexpression vector pcs2(+). The ZIP1 3′ UTR including the binding site of miR-24b-3p was amplified and inserted into the pmir-GLO vector (Promega, Madison, WI, USA) at the 3′ end of the *luc2* (*firefly luciferase*) gene (pmir-GLO-ZIP1-3′ UTR). Similarly, the vectors of pmir-GLO-lnc017705 were obtained using the same method. Primer sequences were shown in Supplementary Table 1. All constructs were verified by sequencing.

### Overexpression Assay

When the cell confluence reached about 80%, the pcs2+-miR-24b-3p and pcs2+-lnc017705 were transfected into cells, which were derived from the connective tissue surrounding IB in *M. amblycephala* and cultured in our laboratory using Lipofectamine 2000 (Invitrogen, USA). The relative expressions of miR-24b-3p, lnc017705, and ZIP1 were tested following the above method.

### Luciferase Activity Assay

When the cell confluence reached about 80%, the pcs2+-miR-24b-3p, pcs2+-lnc017705, and pmir-GLO-ZIP1-3′ UTR were co-transfected into HEK293T cells. Similarly, the pcs2+-miR-24b-3p and pmir-GLO-lnc017705 were co-transfected into cells. After incubation for 24 h, the cells were collected and lysed, and dual luciferase activity was measured using a dual luciferase assay kit (Vazyme, Nanjing, China) and an automatic microplate reader (Molecular Devices, Sunnyvale, USA). The firefly luciferase activity was normalized against Renilla luciferase activity.

## Results

### Correction and Mapping of SMRT Sequencing

Of 326,088 generated CCS, FLNC reads from SMRT sequencing have a total number of 278,126 with a mean length of 2,543 bp (Table 1). Through the rigorous screening process, a total of 179,990 correct consensus reads with a mean length of 2,481 bp and N50 of 3,310 bp were generated. According to the mapping results, the sequences are classified into four types: unmapped (2.34%), multiple mapped (9.06%), reads mapped to a sense strand “+” (46.39%), reads mapped to an antisense strand “–” (42.21%) (Supplementary Figure 1A). The mapping coverage and identity of each transcript and density of FLNC sequences in chromosomes showed that transcripts with 98–100% coverage accounted for 75%, the mapping identification of which was 100% (Supplementary Figures 1B,C).

**Table 1:** Summary of reads from PacBio Iso-Seq.

	**Subread**	**CCS**	**FLNC**	**ICE consensus**	**Polished consensus**	**Correct consensus**
Number	12,394,619	326,088	278,126	180,043	179,990	179,990
Mean length (bp)	1,739	2,699	2,543	2,463	2,479	2,481
N50 (bp)	2,406	3,417	3,293	3,296	3,310	3,310

### Gene Structure Analysis and Identification of LncRNA From SMRT Sequencing

**Figure 2:**
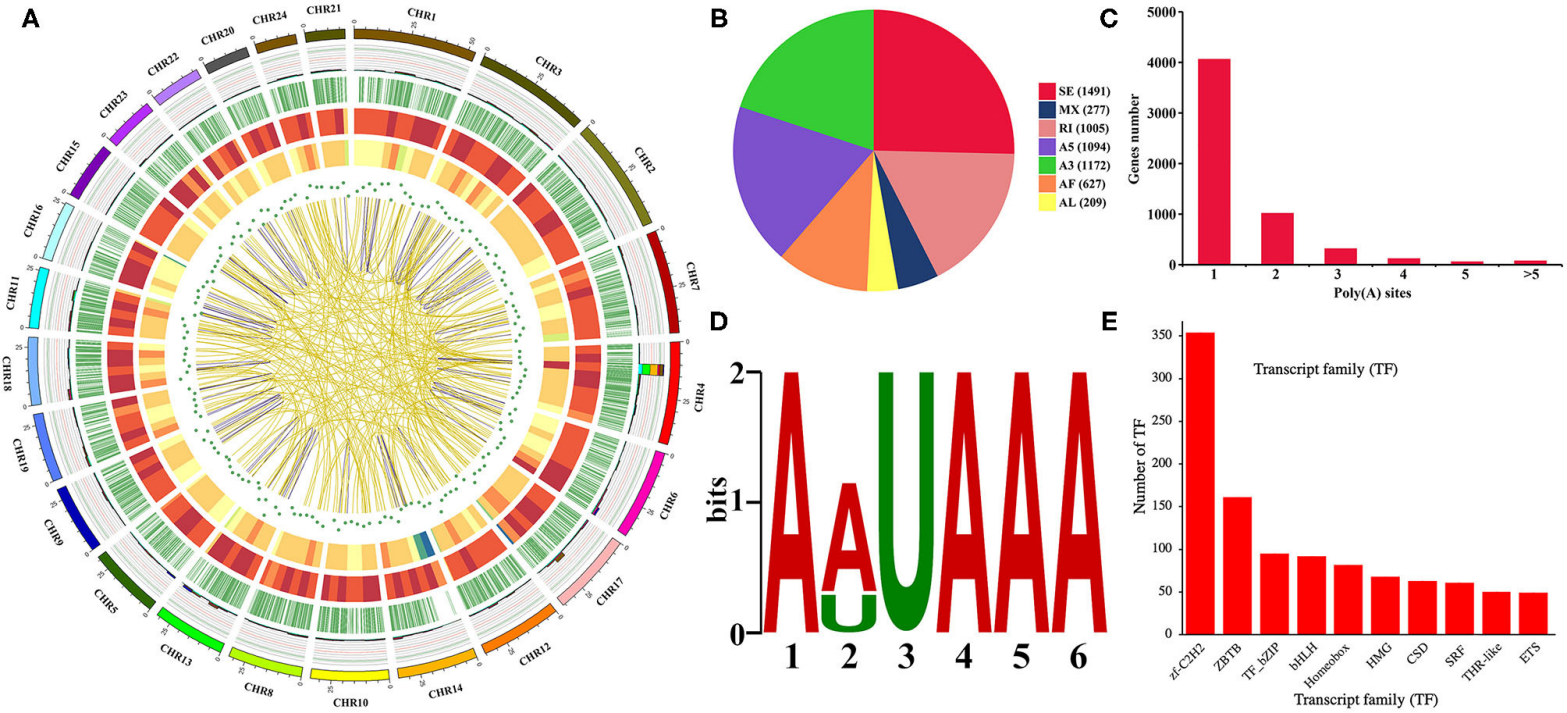
Gene structure analysis. **(A)** Circos visualization of Iso-Seq data at the genome-wide level. From the outside to the inside: 1. Chromosome sequence. 2. Accumulation histogram of alternative splicing events (light blue indicates RI, green indicates A3, yellow indicates A5, purple indicates SE, red indicates MX, brown indicates AF, and dark blue indicates AL). 3. Alternative polyadenylation sites. 4. Distribution of novel transcripts from Iso-Seq. The color closer to red represents higher density, and the color closer to blue represents lower density. 5. Distribution of novel genes from Iso-Seq. The color closer to red represents higher density. 6. LncRNA density. The dot closer to the center represents lower density. 7. Linkage of fusion transcripts. Purple indicates intrachromosomal, and yellow indicates interchromosomal. **(B)** Statistics of alternative splicing events. **(C)** Statistics of alternative polyadenylation events. **(D)** MEME analysis of motif upstream of the poly(A) site. **(E)** Transcription factor analysis.

A total of 34,544 isoforms with a mean length of 2,537 bp and a N50 of 3,258 bp were obtained, including 2,209 isoforms of known genes, 26,302 novel isoform of known genes, and 6,033 isoforms of novel genes (Supplementary Figure 2A). The results also indicated that Iso-Seq generates longer transcripts and more gene isoforms than the reference genome (Supplementary Figures 2B–D). The density distribution of new genes and transcripts on chromosomes were analyzed and shown in Figure 2A. The SUPPA analysis revealed that SE is the most abundant AS event followed by A3, A5, and RI events (Figures 2A,B). In order to identify poly(A) sequence signals in our PacBio Iso-Seq data, a MEME analysis was performed on the sequence of 50 nucleotides upstream of the 8,541 poly(A) sites in 5,639 genes (Figures 2A,C). The conserved motif (AAUAAA) was identified upstream of poly(A) cleavage sites (Figure 2D). According to the Pfam file of the TF family, a total of 1,462 genes belonging to 59 TF families were identified using hmmsearch (Figure 2E). Besides, a total of 31,186 complete ORFs as well as 17,901 5′ and 27,602 3′ UTR sequences of the isoforms were predicted. And a total of 4,458 fusion transcripts were identified in the present study, whose linkage on intra-chromosomes and inter-chromosomes was shown in Figure 2A. After prediction of coding potential, transcripts without coding potential are defined as our candidate set of lncRNAs (Supplementary Figure 3A), which had a total number of 5,199 and were divided into four groups based on their position in the genome: long intergenic non-coding RNA (lincRNA), antisense, sense intronic, and sense overlapping (Supplementary Figure 3B). Besides, the results showed that the mean length of lncRNAs was longer than that of mRNAs, and most lncRNAs only had one exon (Supplementary Figures 3C,D).

### Expression Profiles of IB Growth-Dependent lncRNA and mRNA

**Figure 3:**
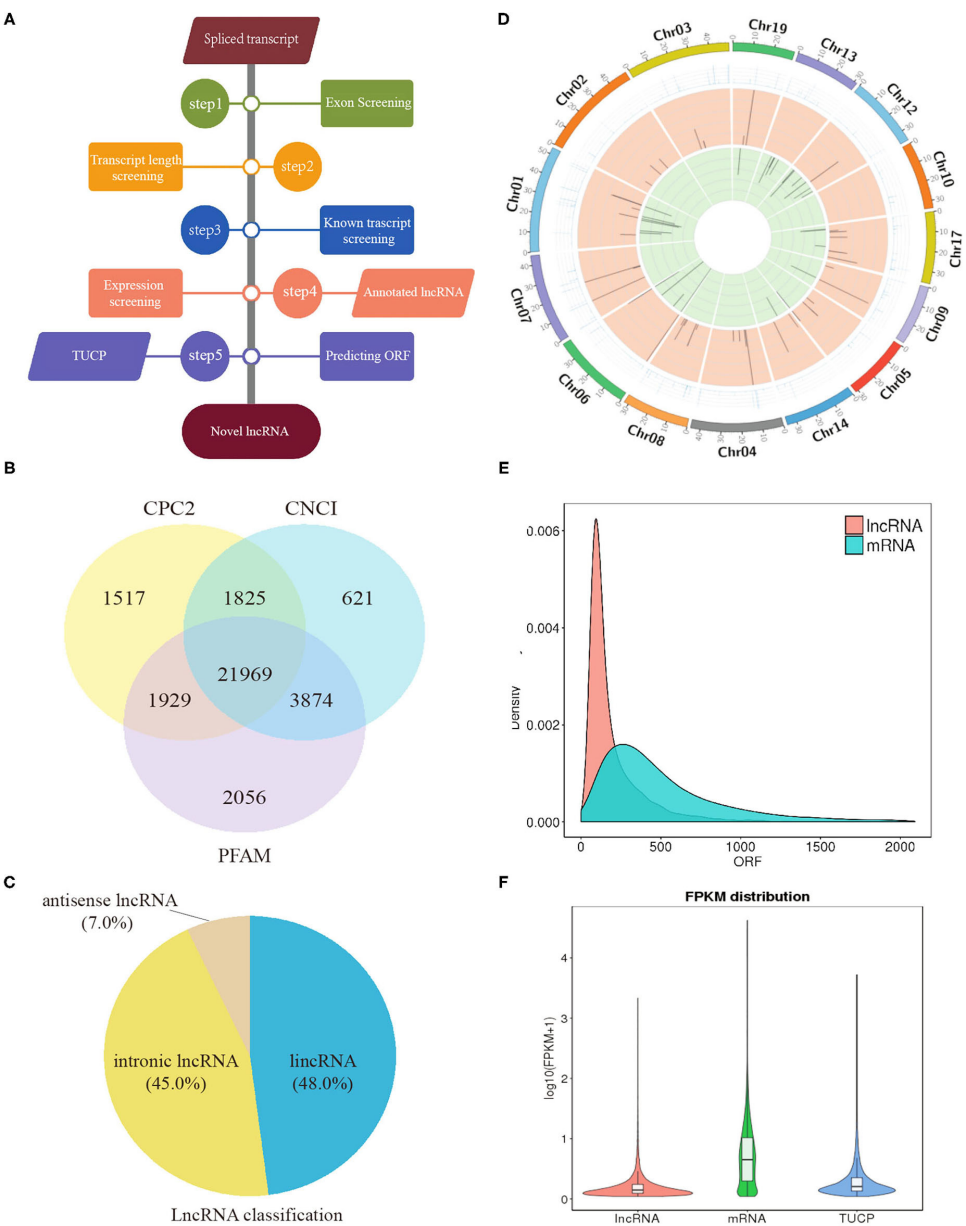
Identification of lncRNA in intermuscular bones. **(A)** Workflow for the preparation and analysis of lncRNA libraries. **(B)** Venn diagram analysis of lncRNAs identified from three software. **(C)** The classification of lncRNAs. **(D)** The distribution of lncRNAs in different chromosomes: the outermost circle is the chromosome; the second circle is the FPKM value of the samples on the corresponding chromosome; the third and fourth circles are the distribution of the significantly upregulated genes and the significantly downregulated genes in IB_III compared to IB_I on the chromosome, respectively. **(E)** Density distribution of lncRNA and mRNA with different ORF lengths. **(F)** Violin diagram of the expression of lncRNA, mRNA, and TUCP transcripts.

**Figure 4:**
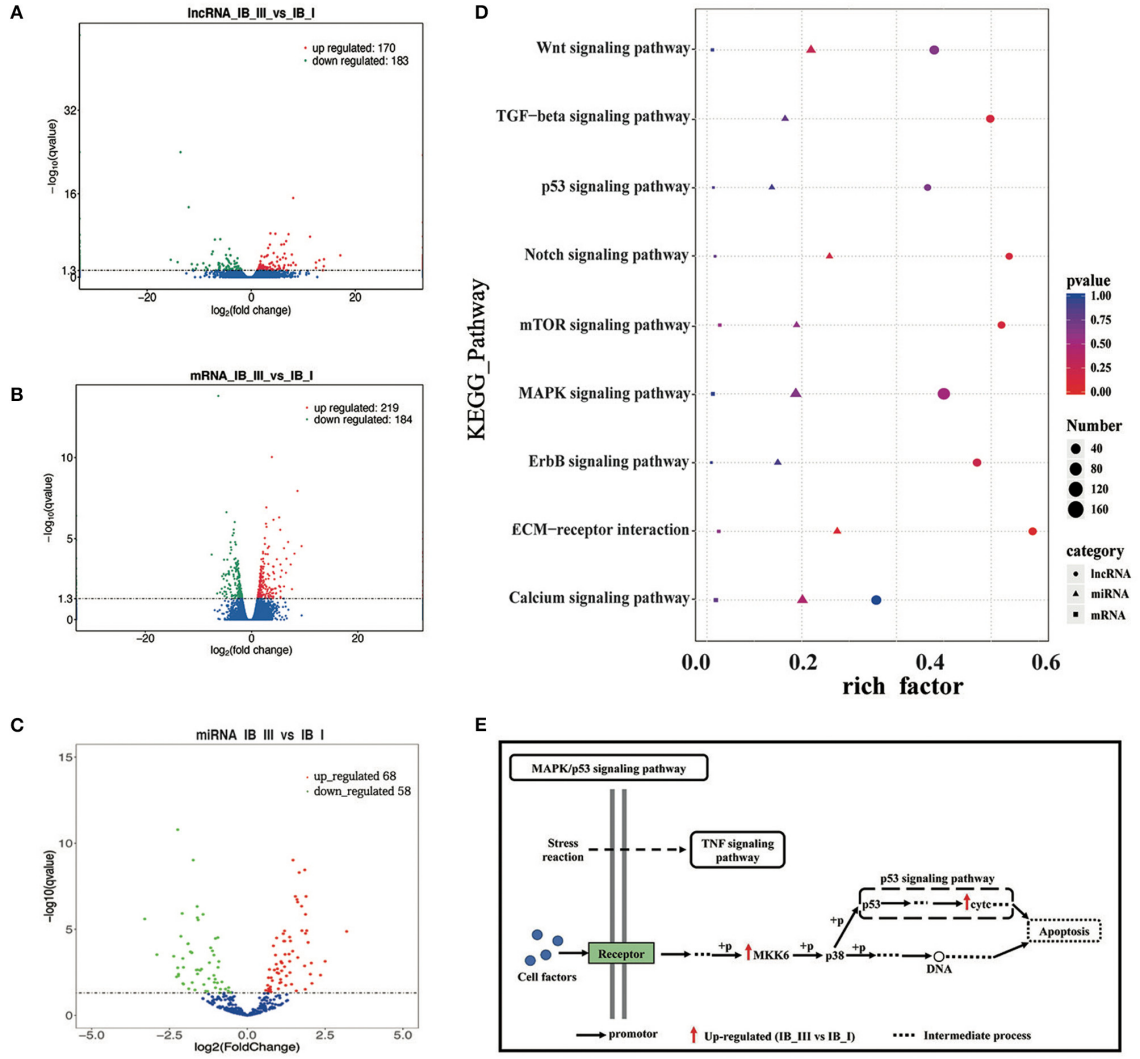
The analysis of differential expression and KEGG enrichment of lncRNA, mRNA, and miRNA in IB_I and IB_III. **(A–C)** Volcano map of differentially expressed lncRNA, mRNA, and miRNA. **(D)** The KEGG pathways enriched by lncRNA, mRNAand miRNA, relating to bone development. The color and size of shape represent *p*-value and enrichment numbers, respectively; the circle, triangle and square represent lncRNA, mRNAand miRNA, respectively. **(E)** The schematic diagram of MAPK/p53 signaling pathway. The black arrow represents the promotor; the red arrow represents the up-regulated (IB_III vs IB_I); the dotted line represents the intermediate process.

In total, clean reads ranging from 83,654,272 to 91,891,080 and 87,038,458 to 95,445,674 were generated from IB_I and IB_III libraries, respectively, with three replicates. Of these, 84.59–87.58% reads in IB_I and 85.69–87.03% reads in IB_III can be mapped to the reference genome (Supplementary Table 2). The proportion of clean reads aligning uniquely to the reference sequence ranged from 78.83 to 81.92% in six libraries. According to the workflow as shown in Figure 3A, a total of 21,969 candidates lncRNAs were identified in IB libraries (Figure 3B), while the percentages of lincRNA, antisense lncRNAs, and intronic lncRNAs were 48.0, 7.0, and 45.0%, respectively (Figure 3C). The distribution pattern of differently expressed lncRNAs on different chromosomes was shown in Figure 3D. As illustrated in Figure 3E, the mean length of lncRNAs was shorter than that of protein-coding transcripts. In order to analyze the expression of different transcripts including lncRNAs, mRNAs, and transcripts of uncertain coding potential (TUCPs), StringTieeB software was used, and the result showed that the expression level of mRNA was higher than the expression level of lncRNA and TUCP (Figure 3F). Based on the expression level comparison, we found that 353 lncRNAs and 403 mRNAs were significantly differentially expressed between IB_I and IB_III (*P* < 0.05), respectively (Figures 4A,B; Supplementary Tables 3, 4). The hierarchical clustering analysis of their expression levels along with the Pearson correlation analysis was conducted and shown in Supplementary Figures 4A–C. Compared with those in IB_I, 170 and 183 lncRNAs were upregulated and downregulated in IB_III, respectively, while 219 and 184 mRNAs were upregulated and downregulated with an expression level fold change of more than 2 (Figures 4A,B). Besides, LNC_020948 (XLOC_153030) and MamblycephalaGene17723 (*act2*) were the most significantly differentially expressed lncRNA and mRNA between IB_III and IB_I, respectively. Among the lncRNAs, LNC_000059 (XLOC_007806) was the most highly expressed in IB_I, followed by LNC_020433 (XLOC_14908) and LNC_02798 (XLOC_159148), while the LNC_021798 (XLOC_159148) showed the highest expression in IB_III, followed by LNC_006232 (XLOC_052479) and LNC_004556 (XLOC_052479).

### Expression Profiles of IB Growth-Dependent miRNA

After discarding low-quality reads, 3′ and 5′ adaptors and sequences with <18 nt, 19,553,745, 21,510,084, 25,513,637, 118,755,553, 22,297,421, and 19,168,240 clean reads were obtained from six libraries (Supplementary Table 5). Little differences in the length distribution of the sequences were identified among the six libraries. Most of the small RNAs were 21–23 nt in length, with 22 nt being the most abundant length (>40%) in all libraries. The total number of unique reads in IB_I and IB_III libraries ranged from 18,289,440 to 23,841,205 and 17,744,474 to 20,789,160, respectively. After reference sequence mapping, 16,999,090 (92.94%), 18,418,061 (92.53%), 21,712,011 (91.07%), 16,645,236 (93.81%), 19,471,908 (93.66%), and 16,694,103 (93.10%) mapped unique sRNAs were then used for miRNA identification and ncRNA annotation (Supplementary Table 5). The identification of the known miRNAs and novel miRNAs was accomplished by mapping the unique sequences in miRBase 20. The results showed that a total of 304 mapped unique miRNAs were detected and that 204 miRNAs were identified as novel in this study (Supplemental Table 6). After differential analysis, 126 miRNAs were identified with differential expression (*P* < 0.05) in IB_I and IB_III, including 68 upregulated and 58 downregulated miRNAs (Figure 4C). Of these differentially expressed miRNAs, the expression level of dre-miR-190b has the most significant difference and dre-miR-152 and dre-miR-99 had the highest expression levels in IB_I and IB_III, respectively (Supplementary Table 7).

### GO and KEGG Analyses

We performed GO enrichment analysis to further investigate the potential functions of differentially expressed lncRNAs, miRNAs, and mRNAs in regulating IB development. As a result, GO enrichment of target genes of differentially expressed lncRNAs was categorized into 4,081 function groups (*P* < 0.05, Supplementary Table 8), while the GO enrichment of differentially expressed mRNAs was categorized into 1,520 function groups (*P* < 0.05, Supplementary Table 9). The GO function annotations of lncRNAs are shown in Supplementary Figure 5A. In addition, our data showed that the targets of differentially expressed lncRNAs and differentially expressed mRNAs were enriched into 158 and 92 pathways (*P* < 0.05, Supplementary Tables 10, 11), respectively. The top 20 enriched KEGG pathways of lncRNAs and mRNAs were presented in Supplementary Figures 6A,B. The miRNAs can regulate the expression of target genes; thus, differentially expressed target mRNA gene enrichment analysis can substitute for enrichment analysis of differentially expressed miRNA. A total of 3,517 GO term groups and 150 KEGG term groups of mRNAs (*P* < 0.05, Supplementary Tables 12, 13) targeted by differentially expressed miRNAs were enriched, while the enrichments and the top 20 KEGG pathways were shown in Supplementary Figures 5B,C, respectively. Based on the pathway analysis, the differentially expressed lncRNAs/miRNAs/mRNAs were commonly involved in eight signaling pathways related to bone development, including the Wnt signaling pathway, MAPK signaling pathway, p53 signaling pathway, mTOR signaling pathway, extracellular matrix (ECM)–receptor, ErbB signaling pathway, calcium signaling pathway, and Notch signaling pathway (Figure 4D). In these pathways, the differentially expressed genes (*map2k6* and *cytc*) in the MAPK/p53 signaling pathway were significantly upregulated, and the differentially expressed genes (*lama3* and *thbs4b*) in the ECM–receptor pathway were significantly downregulated. The pattern diagram of the MAPK/p53 signaling pathway was shown in Figure 4E.

### Construction of the ceRNA Network

**Figure 5:**
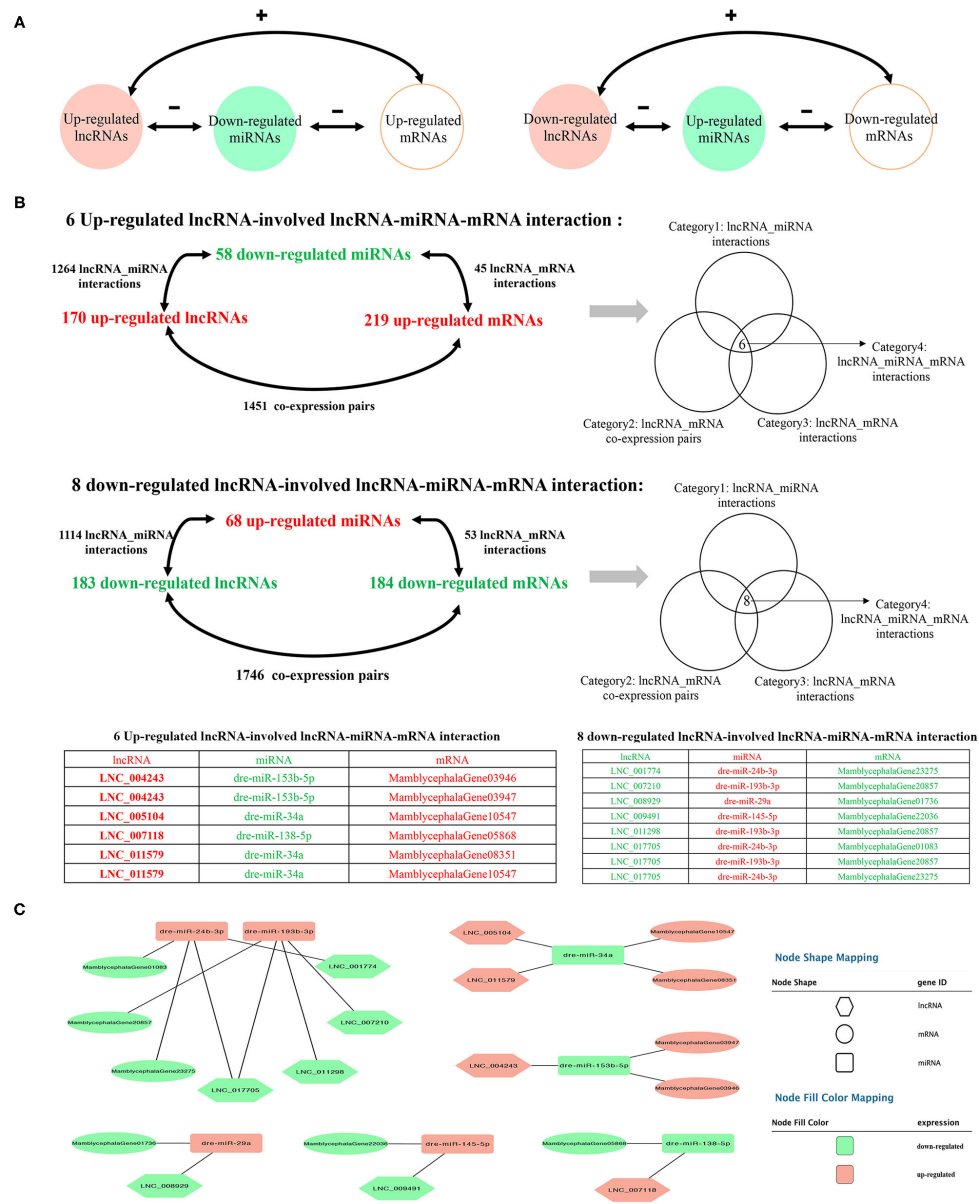
Identification of the ceRNA regulatory network. **(A)** The schematic diagram of lncRNA–miRNA–mRNA axes. “–” represents the negative correlation of the expression. “+” represents the positive correlation of the expression. **(B)** The filtering and categories of lncRNA–miRNA–mRNA interactions. **(C)** The cytoscape diagram of lncRNA–miRNA–mRNA interactions.

To further explore the potential lncRNA–miRNA–mRNA regulatory network, the co-expression analysis of differentially expressed lncRNAs and mRNAs between IB_I and IB_III was conducted firstly. A total of 3,197 co-expression lncRNA–mRNA pairs were obtained (*r* > 0.95 and *P* < 0.001) (Supplementary Table 14), and 2,053 lncRNA–miRNA–mRNA targeted pairs were generated (Supplementary Table 15). A total of 14 ceRNA pairs was obtained by merging the co-expression pairs and lncRNA–miRNA–mRNA targeted pairs, in which six upregulated lncRNAs and eight downregulated lncRNAs were involved in the ceRNA pairs. The detailed prediction process was shown in Figure 5, and the ceRNA regulatory network was visualized using Cytoscape software. Of these, we found that dre-miR-24b-3p and dre-miR-193b-3p were the core elements of the ceRNA pairs, interacting with four lncRNAs and three mRNAs, including LNC_007210, LNC_011298, LNC_001774, LNC_017705, MamblycephalaGene01083 (ZIP1), MamblycephalaGene20857 (C6), and MamblycephalaGene23275 (Figures 5B,C).

### Validation of Transcriptome Sequencing

**Figure 6:**
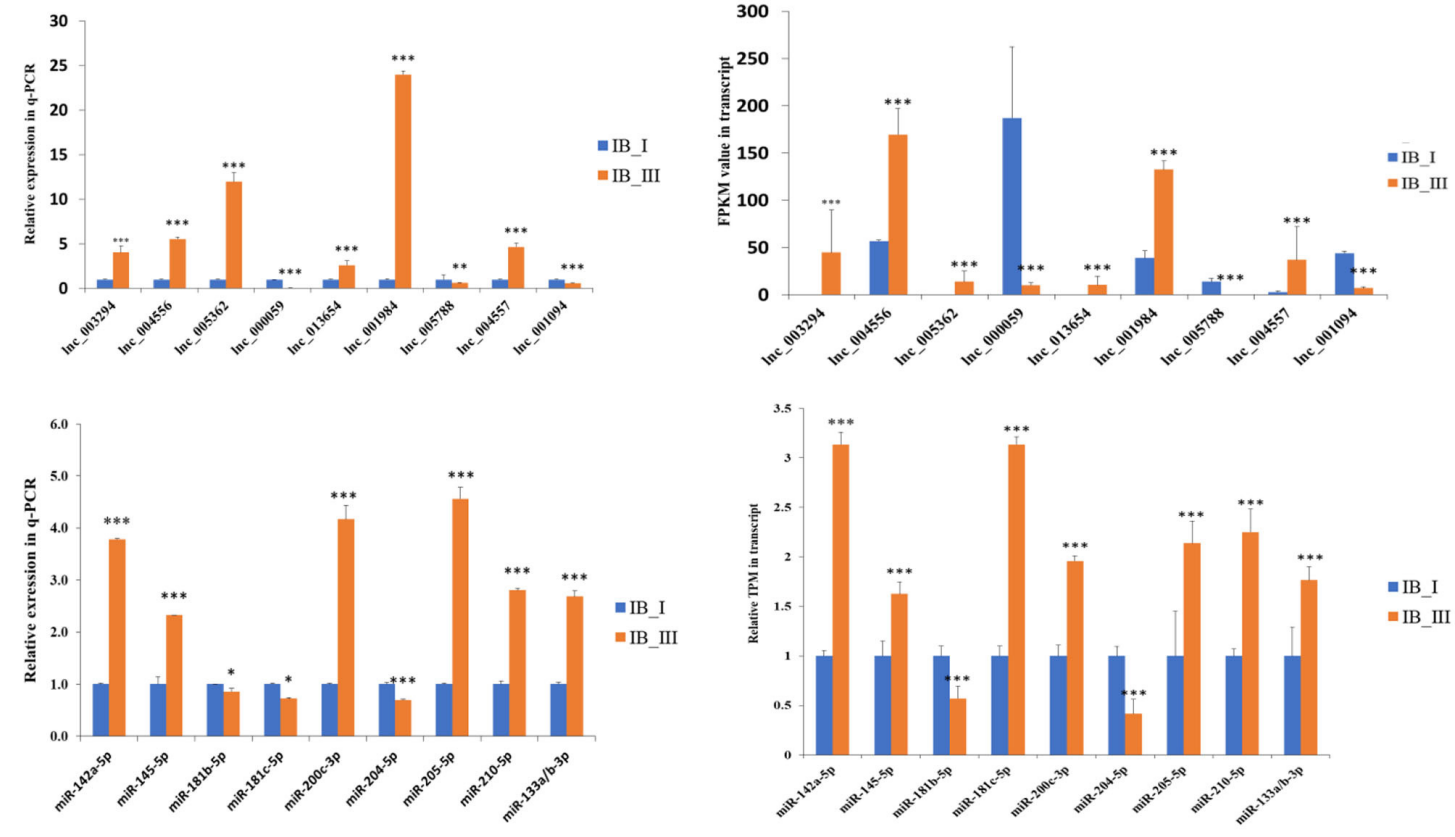
The expression comparison of lncRNAs and miRNAs in RNA-Seq and qRT-PCR. The significant difference was presented as *P < 0.05, **P < 0.01, and ***P < 0.001.

The gel electrophoresis separation results showed that all five genes have different amounts of isoforms (Supplementary Figure 2E), which indicated the reliability of the data generated in the present study and the different expression levels of different transcript isoforms. For example, in Gene15055, the expression of isoform3 is obviously higher than that of isoform1 and isoform2 (Supplementary Figure 2E). The qPCR results showed that the expression patterns between IB_I and IB_III in *M. amblycephala* were consistent with the RNA-Seq results (Figure 6). The qPCR and RNA-Seq results of nine lncRNAs and nine miRNAs in the two periods also showed similar expression changes (Figure 6).

### Lnc017705 Acts as a ceRNA for miR-24b-3p

**Figure 7:**
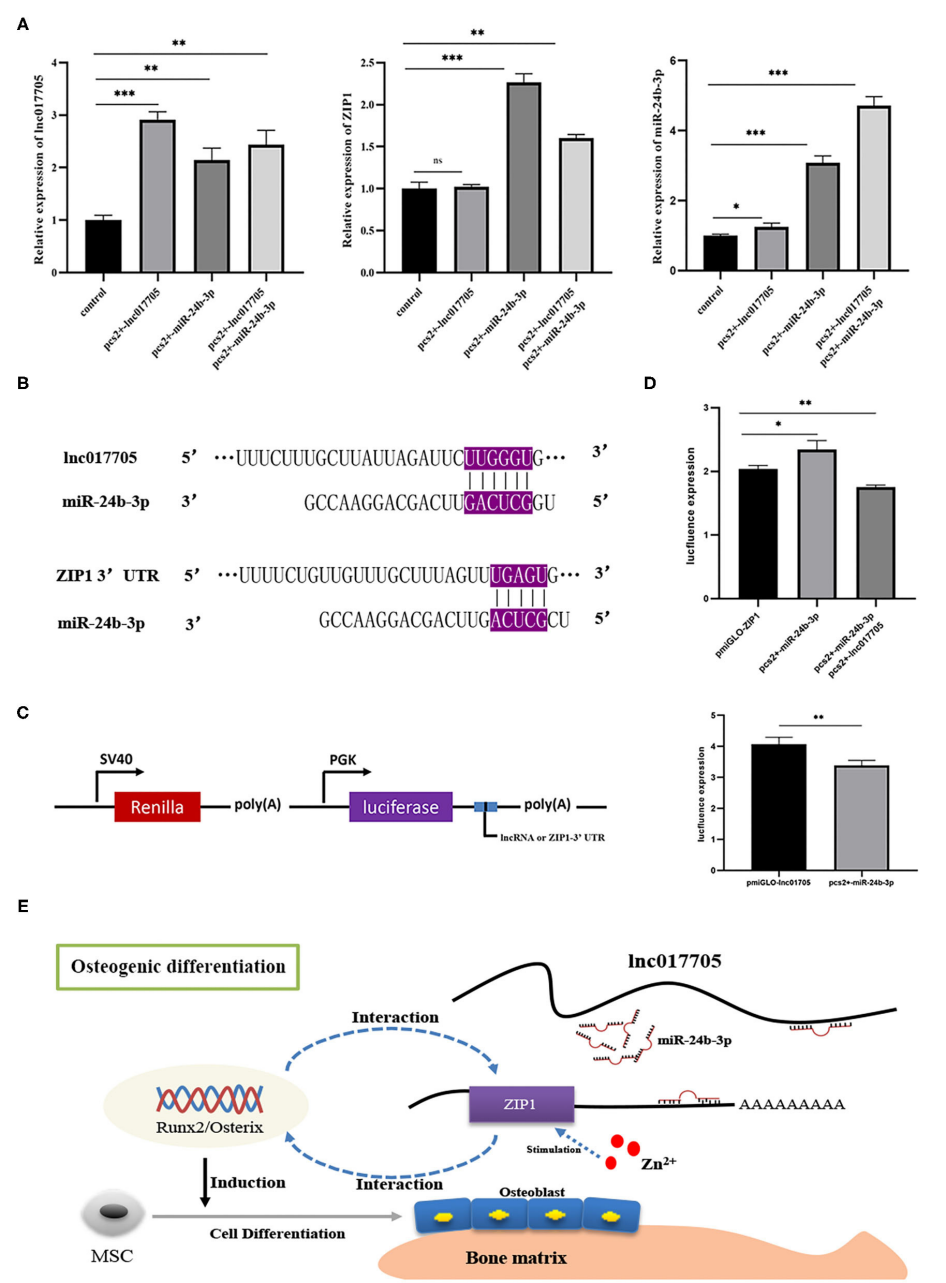
The results of overexpression experiments and dual luciferase reporter gene of the lnc017705/miR-24b-3p/ZIP1 axis. **(A)** The result of lnc017705 and miR-24b-3p overexpression. **(B)** Targeting relationship prediction between miR-24b-3p and lnc017705 as well as ZIP1. **(C)** The construction of the dual luciferase vector. **(D)** The target verification of lnc017705 or ZIP1 with miRNA. **(E)** A regulatory schematic diagram of the lnc017705/miR-24a-3p-ZIP1 axis in osteogenic differentiation. *p < 0.05, **p < 0.01, ***p < 0.001.

Given that ZIP1 can promote osteogenic differentiation by regulating *runx2* and *osterix* expressions (Fu et al., 2018), we further explored the interaction mechanism of the regulatory axis lnc017705–miR-24b-3p–ZIP1 identified from the ceRNA network (Figures 5B,C). We transfected pcs2+-lnc017705 into connective tissue cells and found that lncRNA overexpression led to a 3-fold increase of lnc017705 expression (Figure 7A). The increasing lnc017705 could induce the expression of endogenous miR-24b-3p, and the pcs2+-miR-24b-3p transcription was promoted when co-transfecting pcs2+-lnc017705 and pcs2+-miR-24b-3p (Figure 7A). On the other hand, miR-24b-3p overexpression caused a significant increase in the expressions of lnc017705 and ZIP1 (Figure 7A). After co-transfection of pcs2+-lnc017705 and pcs2+-miR-24b-3p, the lncRNA expression increased compared with that only transfected by miR-24b-3p but lower than that only transfected by pcs2+-lnc017705 (Figure 7A). To determine the targeted relationship between lnc017705 and miR-24b-3p, we generated a miR-24b-3p sensor by inserting the lnc017705 sequence downstream of the *luc2* gene in the pmir-GLO vector (Figure 7C). We found that miR-24b-3p markedly decreased the *luc2* activity of pmir-GLO-lnc017705 in HEK293 cells (Figure 7D), which determined the target relationship between lnc017705 and miR-24b-3p.

Our bioinformatics analysis results show that ZIP1 is a potential target gene of miR-24b-3p (Figure 7B). Meanwhile, we found that ZIP1 mRNA expression could be rescued by co-transfection of pcs2+-lnc017705 and pcs2+-miR-24b-3p, compared with that only transfected by pcs2+-miR-24b-3p (Figure 7B). To eliminate the influence of miR-24b-3p on ZIP1, a dual luciferase activity assay was performed, referring to lnc017705 and miR-24b-3p. The results showed that miR-24b-3p could significantly increase *luc2* activity when co-transfected with pcs2+-miR-24b-3p and pmir-GLO-ZIP1-3′ UTR, but *luc2* activity was decreased after transfecting with additional pcs2+-lnc017705 (Figure 7D).

## Discussion

The multi-omics analysis is an effective way to explore the molecular mechanism underlying complex trait development. In the present study, IB samples were isolated from *M. amblycephala* and used for PacBio library construction, which was then utilized to improve the draft genome annotation and full characterization of the transcriptome. Meanwhile, an integrative analysis of the transcriptome and degradome related to IB growth was presented. Among the KEGG pathways enriched by differentially expressed genes from the transcriptome and degradome of IB, eight KEGG pathways, such as the MAPK signaling pathway, p53 signaling pathway, Wnt signaling pathway, and ECM–receptor interaction pathway, were commonly enriched by differentially expressed lncRNAs, miRNAs, and mRNAs (Figure 4D). This is the first comprehensive overview of multi-omics about fish IB growth.

The MAPK–extracellular signal regulated kinase (ERK) pathway is an important link between the cell surface and nucleus to regulate proliferation and differentiation migration, as well as cell death (Lu et al., 2016). It also plays a critical role in bone formation (Schindeler and Little, 2006). MKK, identified as a mitogen-activated protein kinase (MAPK), can activate p38 protein through phosphorylating a critical -Thr-Gly-Tyr- motif (Goedert et al., 1997; Ono and Han, 2000). Then, phosphorylated p38 can act on the downstream target gene or participate in the p53 signaling pathway to promote cell apoptosis. In the present study, the gene *map2k6* (MamblycephalaGene13655), belonging to MKK families, was upregulated in IB_III and might enhance the activity of p38, followed by promoting cell apoptosis. In the p53 signaling pathway, the electron carrier cytochrome c (*cytc*) was released under certain extreme situations (Kalpage et al., 2020). Once *cytc* is released from the mitochondria into the cytosol, it can interact with the protein apoptosis protease activating factor-1 (*Apaf-1*), which results in the formation of the apoptosome, leading to downstream caspase activation and cell death (Kalpage et al., 2020). Therefore, the significantly upregulated *cytc* (MamblycephalaGene13678) might be the reason for the slow growth of IBs in IB_III. Namely, the activity of cell apoptosis in IB_III was enhanced via upregulation of genes (*map2k6* and *cytc*) involved in the MAPK/p53 signaling pathway.

The ECM consists of a complex mixture of structural and functional macromolecules, including glycosaminoglycans and fibrous proteins (e.g., *collagen, elastin, fibronectin*, and *lammin*) (Timpl, 1996; Van der Flier and Sonnenberg, 2001; Mariman and Wang, 2010), which are usually linked to the cytoskeleton through integrins to control various signaling pathways (Van der Flier and Sonnenberg, 2001). The previous studies have shown that the ECM proteins are associated with biomineralization in the bone tissue (Ravindran and George, 2014; Murshed, 2018) and provide structural and mechanical support for cells (Baroncelli et al., 2018). Therefore, downregulated ECM-related genes (e.g., MamblycephalaGene08032: *lama3* and MamblycephalaGene22942: *thbs4b*) may explain the lower biomineralization activity in IB_III.

ceRNAs can regulate each other at the post-transcription level by competing for shared miRNAs (Qi et al., 2015). For example, lncRNA can indirectly regulate mRNA expression by absorbing endogenous miRNA, which has been introduced in various research fields (Wang R. et al., 2018; Zhang and Lu, 2018). In this study, we had constructed the ceRNA regulator network (lncRNA–miRNA–mRNA) to discover the core regulatory factors related to IB development. A total of 14 ceRNA pairs were generated, composed of 10 lncRNAs, 7 miRNAs, and 10 mRNAs. Among the ceRNA pairs, we found that dre-miR-24b-3p and dre-miR-193b-3p play the role of core elements, interacting with five lncRNAs and three mRNAs, such as lnc017705, MamblycephalaGene01083 (ZIP1), and MamblycephalaGene20857 (C6). As is known to all, bone homeostasis depends on bone resorption and formation by osteoclasts and osteoblasts (Chen et al., 2018), respectively, which are the two main cells participating in bone development (Matsuo and Irie, 2008). Meanwhile, osteoblast and osteoclast formation, differentiation, or apoptosis was affected by functional genes, such as *runx2, osterix, rank, opg*, β*-catenin*, and *zip1* (Anderson et al., 1997; Komori et al., 1997; Nakashima et al., 2002; Boyce and Xing, 2008). Among them, ZIP1, as an essential trace element, is involved in diverse metabolic and signaling pathways (Mohammed et al., 2005) and promotes osteogenic differentiation by forming a zinc–Runx2/Osterix–ZIP1 regulation axis (Fu et al., 2018). The majority of Zn^2+^ in the body was involved in osteogenesis as an activator or coactivator of a variety of proteins such as *runx2* and *osterix* with zinc finger motifs (Yamaguchi et al., 2008; Zhao et al., 2015). Besides, Mohammed et al. (2005) had determined that intracellular zinc content gradually accumulated and further found that ZIP1 was one dominant zinc transporter required for zinc uptake during osteogenesis of mesenchymal stem cells (MSCs). In addition, addition of Zn^2+^ or overexpression of ZIP1 can enhance MSC differentiation and promote the deposition of citrate and Ca^2+^ in mineralized MSCs (Fu et al., 2018). Therefore, it is reasonable to speculate that slow-growth IBs at the IB_III stage might result from decreased osteoblast differentiation and Ca^2+^ deposition caused by the downregulated ZIP1. Meanwhile, miR-24a-3p is the downstream targets of ZIP1 and lnc017705. Considering that miR-24 can regulate osteoblast differentiation of MSCs by targeting Tcf-1 (Nakashima et al., 2002), the lnc017705–miR-24a-3p–ZIP1 regulatory axis is likely to play an important role in the development of IB.

It is well-known that miRNAs inhibit the translation of mRNAs into protein and promote mRNA degradation. In the targeting relationship verification experiment, we found that lnc017705 can bind to miR-24b-3p to influence ZIP1 expression. However, when we singly transfected pcs2+-miR-24b-3p, the expressions of ZIP1 and lnc017705 were increased, which was contrary to expectation. This is because miR-24b-3p produced from pcs2+-miR-24b-3p could relieve the repression of target genes of endogenous miRNAs by available RNA-induced silencing complex (RISC) competition, resulting in upregulation of the corresponding mRNAs and proteins (Khan et al., 2009). This phenomenon was called machinery saturation (Castanotto et al., 2007). Besides, more miR-24b-3ps from pcs2+-miR-24b-3p were produced through co-overexpression of lnc017705 and miR-24b-3p, and ZIP1 expression will decrease since the machinery saturation was destroyed. Thereby, the mRNA level of ZIP1 and *luc2* activity of pmir-GLO-ZIP1-3′ UTR vector were decreased by miRNA oversaturation state. Among numerous ceRNA researches, using miRNA mimics, but not pre-miRNAs (which rely on the nuclear export machinery), becomes the first choice because it can have highly effective inhibition and avoid saturation-related effects (McBride et al., 2008). Despite that, the higher off-targeted rate of miRNA mimics compared with miRNA expression vector is also a problem. So we had provided ideals by using pre-miRNA vector to investigate the ceRNA mechanism in our study. Finally, a summary of the lnc017705–miR-24a-3p–ZIP1 axis in osteogenesis difference is illustrated (Figure 7E), which offers novel clues to elucidate in depth the growth mechanism of teleost IB in future studies.

## Conclusion

This is the first study to reveal the characteristics of the transcriptome and degradome associated with the growth of IBs in fish species. Through pathway enrichment analysis, we found that the expression change of key functional genes between IB_III and IB_I might be the reason for the slow growth of IBs in IB_III. In a word, the activity of cell apoptosis in IB_III was enhanced via upregulated genes (*map2k6* and *cytc*) involved in the MAPK/p53 signaling pathway, and the lower mineralization activity in IB_III may be caused by downregulated genes (*lama3* and *thbs4b*) in the ECM–receptor. Meanwhile, based on targeting relationship verification of lncRNA–miRNA–mRNA ceRNA and expression analyses, it is reasonable to believe that the slow IB growth in IB_III resulted from decreased osteoblast differentiation and Ca^2+^ deposition regulated by the lnc017705–miR-24a-3p–ZIP1 ceRNA network.

## Data Availability Statement

The datasets presented in this study can be found in online repositories. The names of the repository/repositories and accession number(s) can be found at: NCBI, PRJNA544738, and PRJNA640807.

## Ethics Statement

All animals and experiments were conducted in accordance with the Guidelines for Experimental Animals of the Ministry of Science and Technology (Beijing, China). The study was approved by the Institutional Animal Care and Use Ethics Committee of Huazhong Agricultural University. All efforts were made to minimize suffering.

## Author Contributions

Z-XG designed and supervised the study. SW, XD, JD, QLi, QLia, and G-YW performed the experiments. YC and SW analyzed the data and wrote the manuscript. All authors reviewed and approved the manuscript.

## Conflict of Interest

QLi and G-YW were employed by the company Wuhan Xianfeng Aquaculture Technology Co. Ltd, Wuhan, China. The remaining authors declare that the research was conducted in the absence of any commercial or financial relationships that could be construed as a potential conflict of interest.
