# Supplementary material for: Genome-Wide Integrated Analysis Revealed Functions of lncRNA–miRNA–mRNA Interaction in Growth of Intermuscular Bones in Megalobrama amblycephala
Source: Front Cell Dev Biol. 2021 Feb 4;8:603815. doi: 10.3389/fcell.2020.603815 (PMC7891300; doi:10.3389/fcell.2020.603815)
Supplement: Supplementary file 2 [file Data_Sheet_2.docx]

**Genome-wide integrated analysis revealed functions of lncRNA-miRNA-mRNA interaction in growth of intermuscular bones in *Megalobrama amblycephala***

Yulong Chen ^1,2†^, Shiming Wan ^1,2†^, Qing Li ^3^, Xiaoru Dong ^1,2^, Jinghan Diao ^1,2^, Qing Liao ^1,2^, Gui-Ying Wang ^3^, Ze-Xia Gao^1,2,4*^

^1^ College of Fisheries, Key Lab of Agricultural Animal Genetics, Breeding and Reproduction of Ministry of Education/Key Lab of Freshwater Animal Breeding, Ministry of Agriculture, Huazhong Agricultural University, Wuhan, Hubei 430070, China;

^2^ Engineering Research Center of Green development for Conventional Aquatic Biological Industry in the Yangtze River Economic Belt, Ministry of Education, Wuhan 430070, China

^3^ Fisheries Research Institute, Wuhan Academy of Agricultural Sciences/ Wuhan Xianfeng Aquaculture Technology Co. Ltd, Wuhan, China

^4^ Engineering Technology Research Center for Fish Breeding and Culture in Hubei Province, Wuhan 430070, China

^*^Corresponding authors: Ze-Xia Gao; E-mail addresses:[gaozexia@hotmail.com;](mailto:gaozexia@hotmail.com;) [gaozx@mail.hzau.edu.cn;](mailto:gaozx@mail.hzau.edu.cn;) Tel/Fax: +86-27-8728-2113

^†^ These authors contributed equally to this work.

**Supplementary figure legend**

**Figure S1** Results of reference genome mapping. (a) GMAP analysis of corrected reads to reference genome. (b) Coverage and identity distribution of transcripts. (c) The distribution of full-length transcripts on the reference genome. The x-axis indicates the length of a chromosome in millions of bases; the y-axis indicates the density of reads; green indicates “+” strand, red indicates “-” strand

**Figure S2** Classification and characterization of transcripts. (a) Classification of FLNC sequences mapped to reference genome. (b) Distribution of transcript length and density in Iso-Seq data and reference genome. (c) Isoform number of genes in Iso-Seq data and reference genome. (d) Exon number of transcripts in Iso-Seq data and reference genome. (e) Reverse transcription PCR validation of AS isoforms (M: DL2000).

**Figure S3** Identification and analysis of lncRNAs. (a) Venn diagram of lncRNAs predicted by PLEK, CPC, CNCI and Pfam. (b) Classification and proportions of four types of lncRNA. (c) Density and length distributions of mRNAs and lncRNAs. (d) Comparison of exon number of mRNAs and lncRNAs identified in present study.

**Figure S4** The clustered heatmaps volcano maps of lncRNA, mRNA and miRNA in IB_I and IB_III.

**Figure S5** The GO enrichment analysis. (a) The GO enrichment of the co-expression mRNA of differentially expressed lncRNAs. (b) The GO enrichment of the target mRNA of differentially expressed miRNAs.

**Figure S6** Top 20 enriched Kyoto Encyclopedia of Gene and Genome (KEGG) pathways. (a) Top 20 enriched KEGG pathways of the co-expression mRNA of differentially expressed lncRNAs. (b) Top 20 enriched KEGG pathways of differentially expressed mRNAs (c) Top 20 enriched KEGG pathways of the target mRNA of differentially expressed miRNAs.


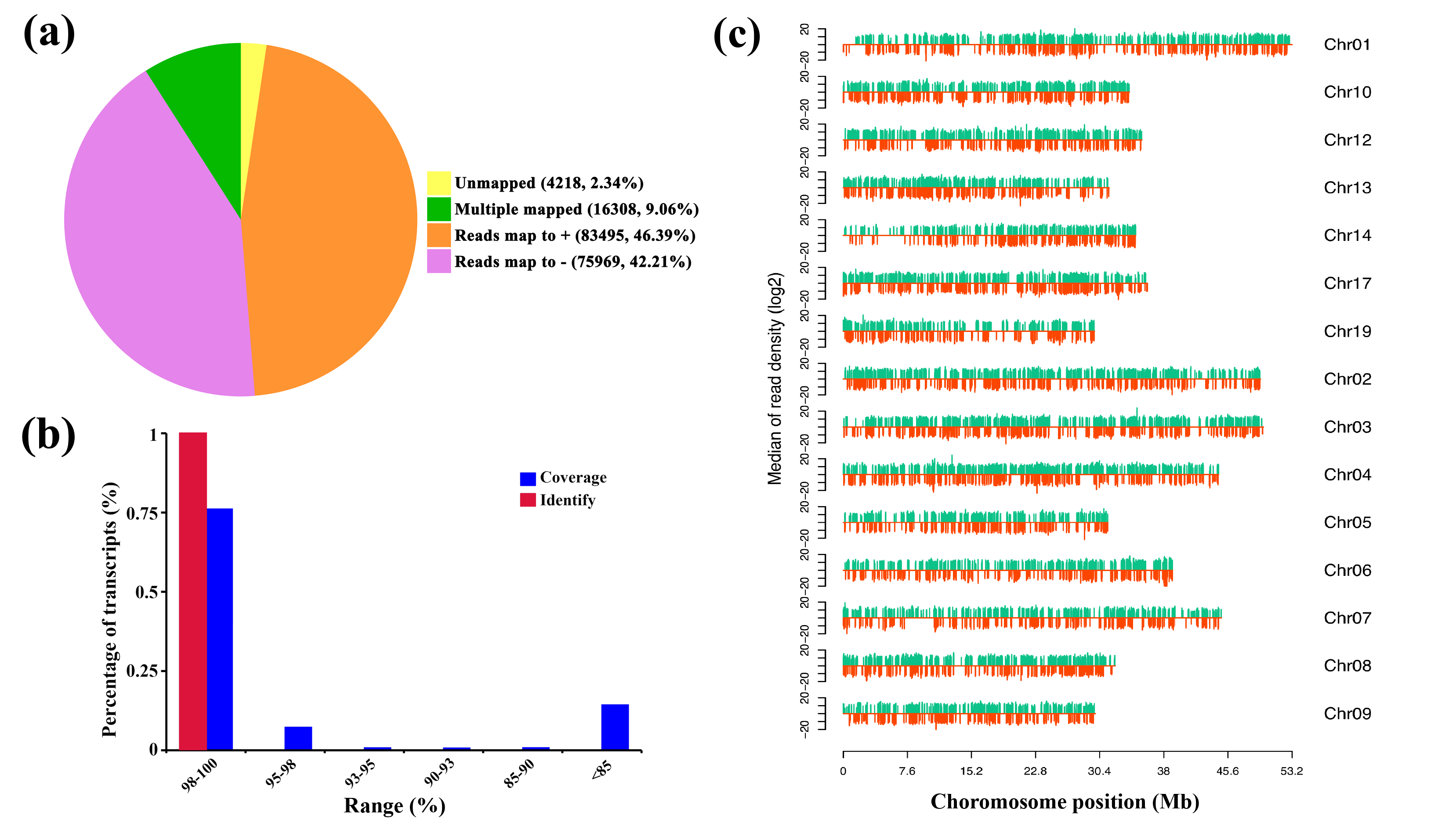


**Figure S1**

**
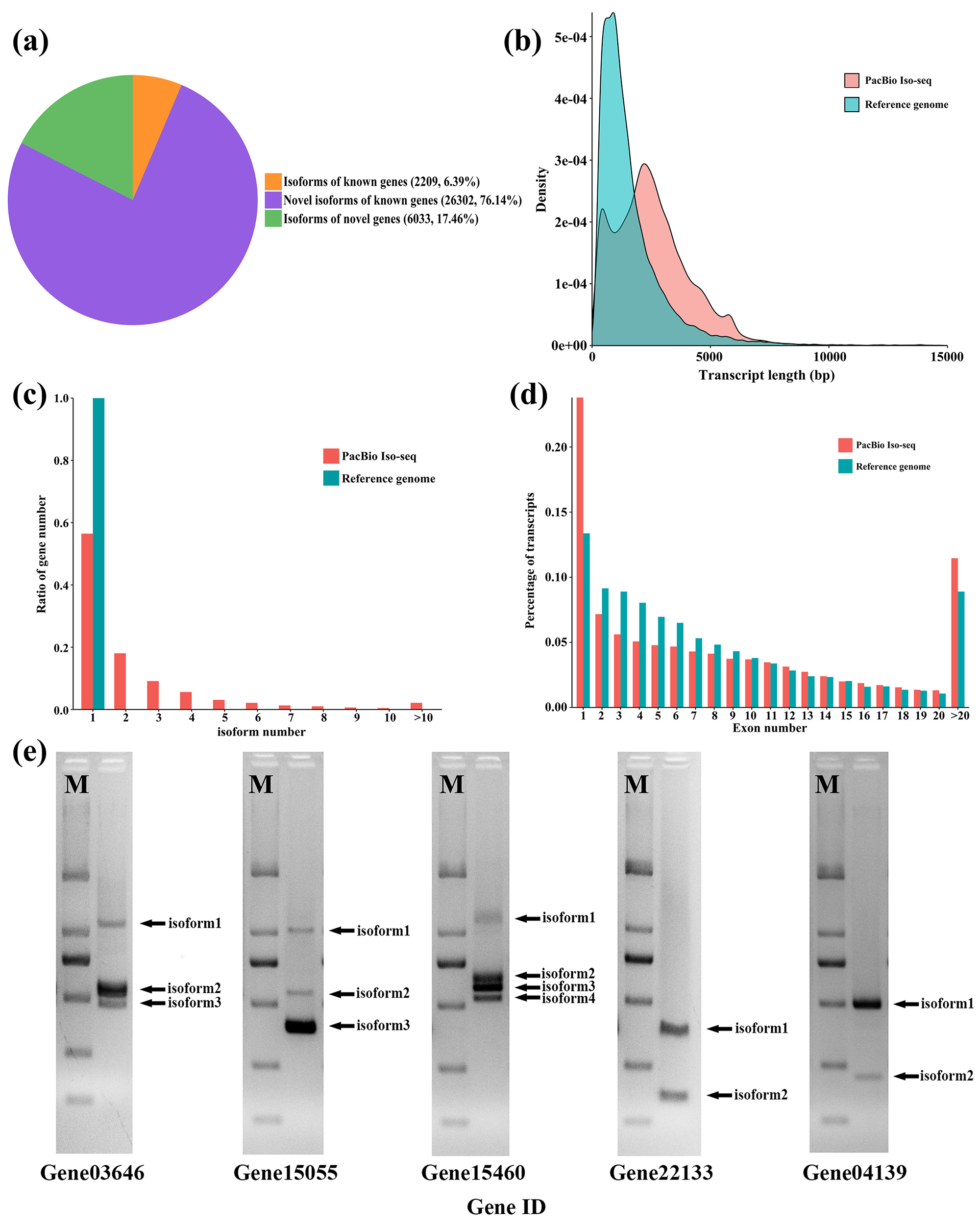
**

**Figure S2**

**
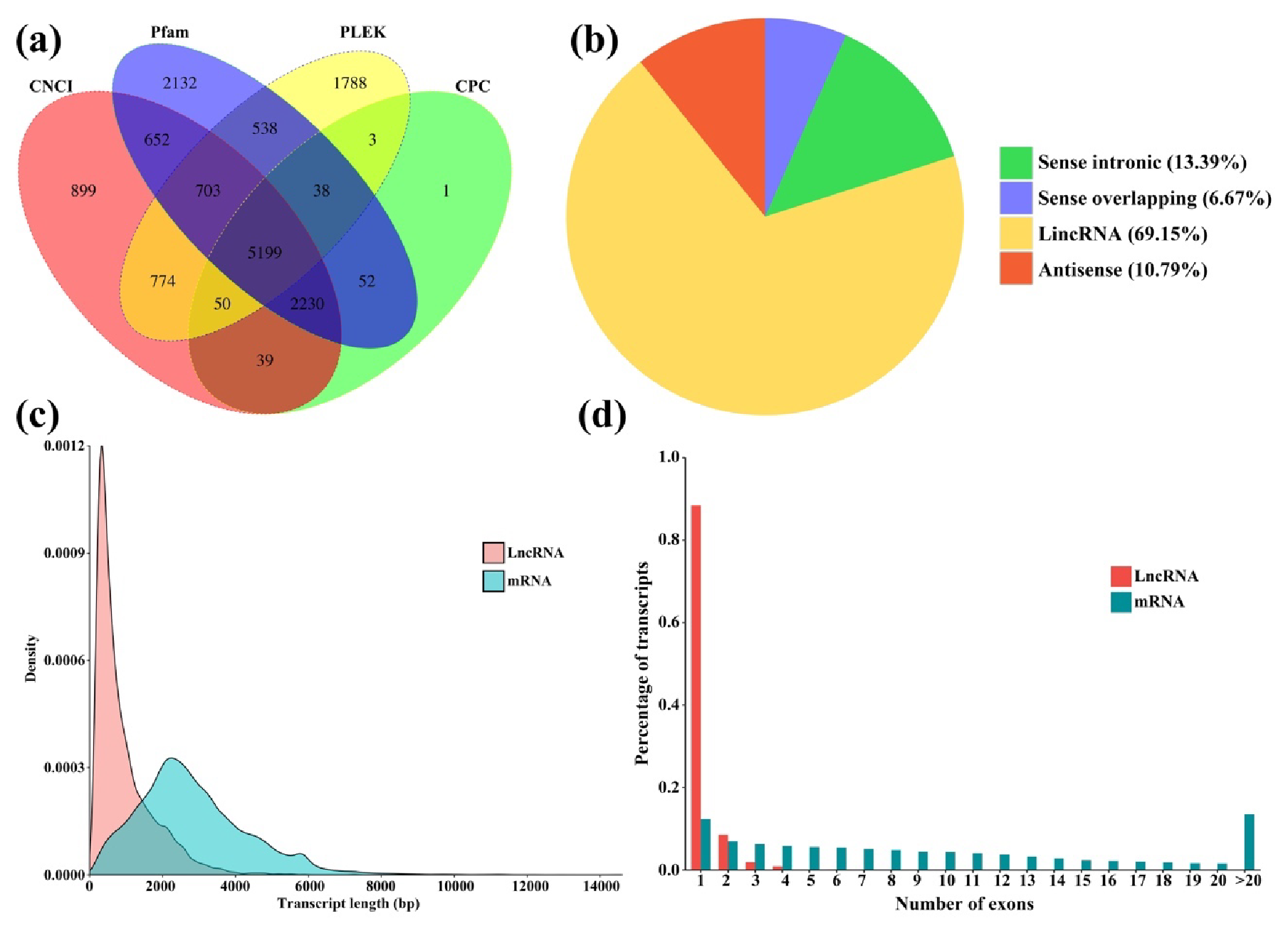
**

**Figure S3**

**
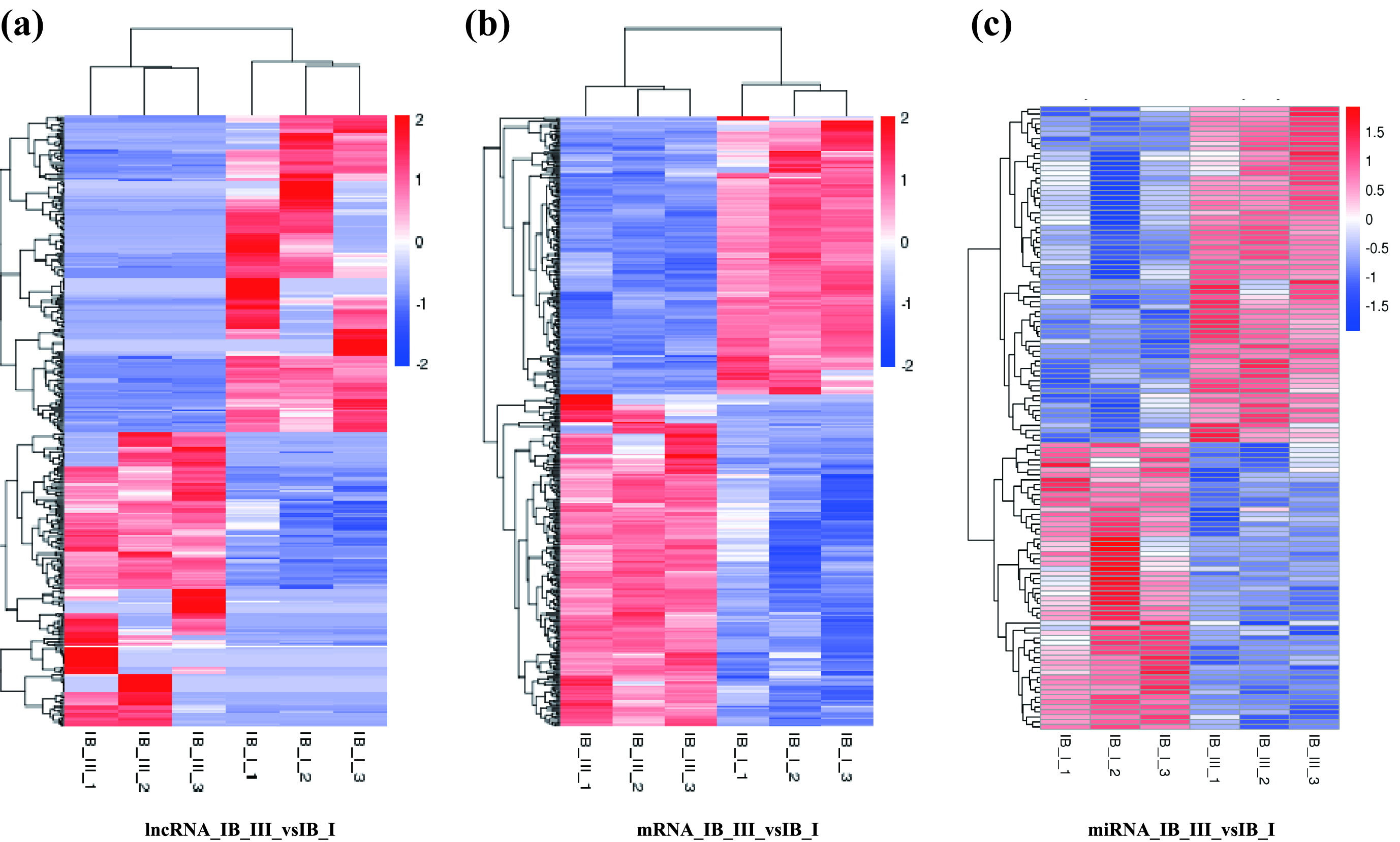
**

**Figure S4**

**
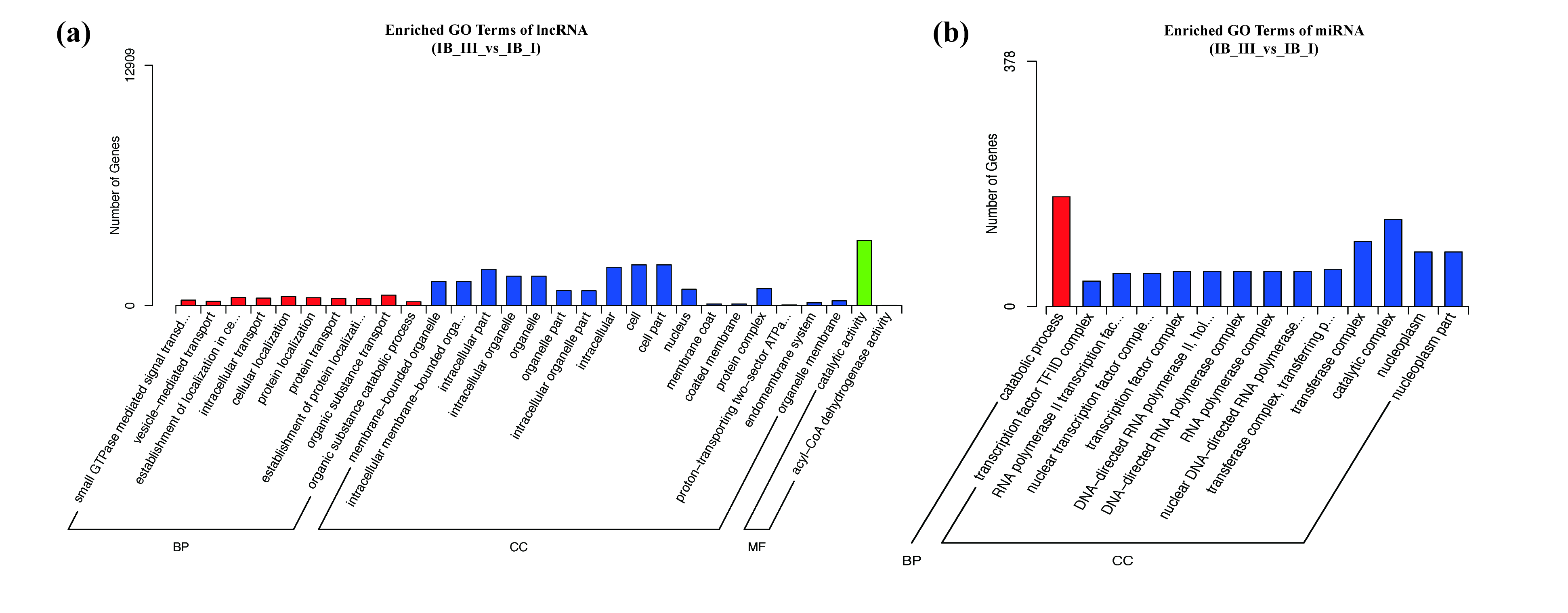
**

**Figure S5**

**
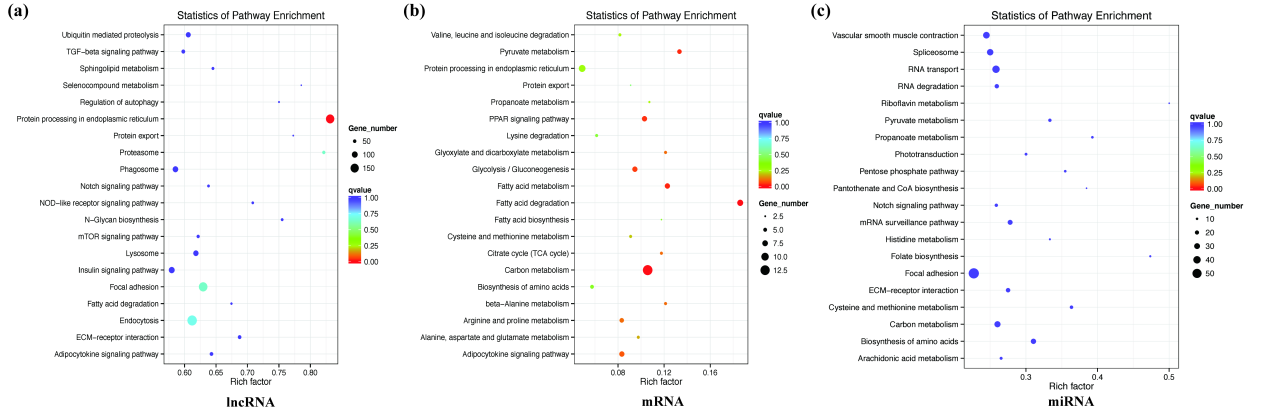
**

**Figure S6**
